# Do Children With Constipation Have Increased Risk of Asthma? Real-World Data From a Nationwide Population-Based Cohort Study

**DOI:** 10.3389/fped.2021.714406

**Published:** 2021-08-30

**Authors:** Yen-Chu Huang, Meng-Che Wu, Yu-Hsun Wang, James Cheng-Chung Wei

**Affiliations:** ^1^Division of Pediatric Gastroenterology, Children's Medical Center, Taichung Veterans General Hospital, Taichung, Taiwan; ^2^Department of Medical Research, Chung Shan Medical University Hospital, Taichung, Taiwan; ^3^Division of Allergy, Immunology and Rheumatology, Chung Shan Medical University Hospital, Taichung, Taiwan; ^4^College of Medicine, Institute of Medicine, Chung Shan Medical University, Taichung, Taiwan; ^5^Graduate Institute of Integrated Medicine, China Medical University, Taichung, Taiwan

**Keywords:** constipation, asthma, longitudinal health insurance database, gut-lung axis, dysbiosis

## Abstract

**Background:** Asthma is one of the most burdensome childhood disorders. Growing evidence disclose intestinal dysbiosis may contribute to asthma via the gut-lung axis. Constipation can lead to alteration of the gut microbiota. The clinical impact of constipation on asthma has not been researched. Therefore, we aim to assess whether pediatric constipation influence the risk of developing asthma by a nationwide population-based cohort study.

**Methods:** We analyzed 10,363 constipated patients and 10,363 individuals without constipation between 1999 and 2013 from Taiwan's National Health Insurance Research Database. Analysis of propensity score was utilized to match age, sex, comorbidities, and medications at a ratio of 1:1. In addition, multiple Cox regression analysis was performed to evaluate the adjusted hazard ratio of asthma. Furthermore, sensitivity tests and a stratified analysis were performed.

**Results:** After adjustment for age, sex, comorbidities, and medications, constipated patients had a 2.36-fold greater risk of asthma compared to those without constipation [adjusted hazard ratio (aHR): 2.36, 95% C.I. 2.04–2.73, *p* < 0.001]. Furthermore, the severity of constipation is associated with an increased risk of asthma; the adjusted hazard ratio was 2.25, 2.85, and 3.44 within < 3, 3–12, and ≥12 times of laxatives prescription within 1 year, respectively (*p* < 0.001).

**Conclusion:** Constipation was correlated with a significantly increased risk of asthma. Pediatricians should be aware of the possibility of asthma in constipated patients. Further research is warranted to investigate the possible pathological mechanisms of this association.

## Introduction

Asthma is one of the most burdensome childhood disorders. It has been estimated that more than 9 million children in the United States (1) and more than 272 million people worldwide have asthma (2). Asthma not only causes social, psychological, and economic burdens, but also has a negative impact on quality of life (3). Currently, there is increasing evidence demonstrating an association between asthma and constipation (4–7), a common situation affecting children globally and a frequent reason for visits to pediatricians. The mean worldwide prevalence of constipation in children is about 12% (8). Although constipation rarely leads to life-threatening complications, it is a cause of physical and psychological distress for children and their families, eventually impairing quality of life and leading to increased health insurance costs.

In recent years, constipation has been shown to be a causative factor in bowel dysbiosis and therapeutic administration has been increasingly applied. Such therapies include probiotics, prebiotics, or synbiotics, which are thought to help regulate the intestinal microbiota (9). Moreover, recent research has shown that the gut microbiota exerts important regulatory effects via the gut-lung axis (10). For instance, dysbiosis (11) and a lower concentration of short-chain fatty acids (SCFAs) (12) in the intestines have been observed in patients with asthma. This condition leads to dysregulation of inflammation (13) and defects in the intestinal epithelial barriers, resulting in increased intestinal permeability (“leaky gut”), which permits the penetration of toxins and microbiome into systemic circulation, thereby activating T helper 2 (Th2) immune responses and then releasing the cytokines such as IL-4, IL-5, IL-9, and IL-13 into systemic circulation, eventually contributing to airway inflammation (14). Some research has suggested that allergic diseases, such as allergic rhinitis (15) and atopic dermatitis (16), might be connected to constipation. Furthermore, previous research has demonstrated that fecal stasis over a long period could influence the microflora and intestinal environment, resulting in deleterious effects on mucosal immunity and intestinal motility (17). It has not been conclusively established that constipation predisposes susceptible people to asthma. Currently, there are still scanty data on the association between constipation and asthma in the literature. Moreover, this association has never been investigated using a large-scale national longitudinal database. We hypothesized that constipation could influence the risk of asthma in children and tested this hypothesis by analyzing a real-world, population-based retrospective cohort from the National Health Insurance Research Database (NHIRD) in Taiwan.

## Methods

### Data Source

This study analyzed data from the National Health Insurance Research Database (NHIRD), which contains the healthcare data of almost 99% of Taiwan's entire population, i.e., ~23 million NHI beneficiaries. The database includes all insurance claims data, including outpatient visits, emergency visits, and hospitalizations. The Longitudinal Health Insurance Database, (LHID) is a subset of the NHIRD comprising one million individuals randomly sampled from the 23 million NHI beneficiaries for the period from 1999 to 2013. The patients' data were de-identified prior to release to the researchers, in accordance with privacy protocols. Written consent from study subjects was not required and waived by the Institutional Review Board of Chung Shan Medical University Hospital (IRB no. CS15134), because the NHIRD comprises de-identified data for research purposes. The study carried out in accordance with IGH-GCP requirements and the essence of Declaration of Helsinki.

### Study Group and Outcome Measurement

The population was composed of patients aged ≤ 18 years old with newly diagnosed constipation (ICD-9-CM codes = 564.0) from 2000 to 2012. To ensure accuracy of diagnoses, we excluded any diagnosis of constipation before 2,000 and only patients with at least three outpatient visits or one hospitalization were selected for inclusion in the final analysis. The index date of this cohort was set as the first date of diagnosis of constipation. Furthermore, to ensure that all individuals had new-onset asthma, we ruled out any diagnosis of asthma (ICD-9-CM = 493) happening before the index date.

The outcome variable was defined as a diagnosis of asthma with at least three outpatient visits or once hospitalization and a prescription for an anti-asthmatic medication including inhaled corticosteroids (ICSs), short-acting β-agonists, and systemic corticosteroids within 1 year (18). The patients were followed up until the occurrence of asthma, 31 December 2013, or withdrawal from the National Health Insurance system, whichever occurred first.

### Covariates and Matching

The baseline characteristics were age, sex, urbanization, allergic rhinitis (ICD-9-CM = 477.9), allergic conjunctivitis (ICD-9-CM = 372.05, 372.14), atopic dermatitis (ICD-9-CM = 691), and bronchiolitis (ICD-9-CM = 466.1). The degree of urbanization was clustered into urban, suburban, and rural by population density (people/km^2^), population ratio of people with college or above educational levels, population ratio of elder people over 65 years old, population ratio of people of agriculture workers and the number of physicians per 100,000 people. The comorbidities were defined as occurring within 1 year prior to the index date with at least three outpatient visits or once hospitalization. In addition, corticosteroids, antihistamines, and antibiotics during the study period were included and defined as usage for at least ≥30 days. To evaluate the severity of constipation, we collected additional information on the total numbers of prescriptions for laxatives [anatomical therapeutic chemical (ATC) classification system code A06A], which were given within 1 year after the index date.

Furthermore, propensity score matching based on age, sex, urbanization, allergic rhinitis, allergic conjunctivitis, atopic dermatitis, bronchiolitis, corticosteroids, antihistamines, and antibiotics was applied to balance the heterogeneity of the two groups. The propensity score was a probability that was estimated through logistic regression. The binary variable was the constipation and non-constipation group.

### Statistical Analysis

Comparisons between the constipation group and non-constipation group were done using absolute standardized differences (ASD). Whenever the ASD was <0.1, the characteristics of the two groups were deemed to be similar (19). Cox proportional hazard model was applied to estimate the hazard ratio of asthma between the constipation and non-constipation groups. Furthermore, Kaplan–Meier analysis was applied to calculate the cumulative incidence of asthma and then log-rank test was used to test the significance.

## Results

**Figure 1:**
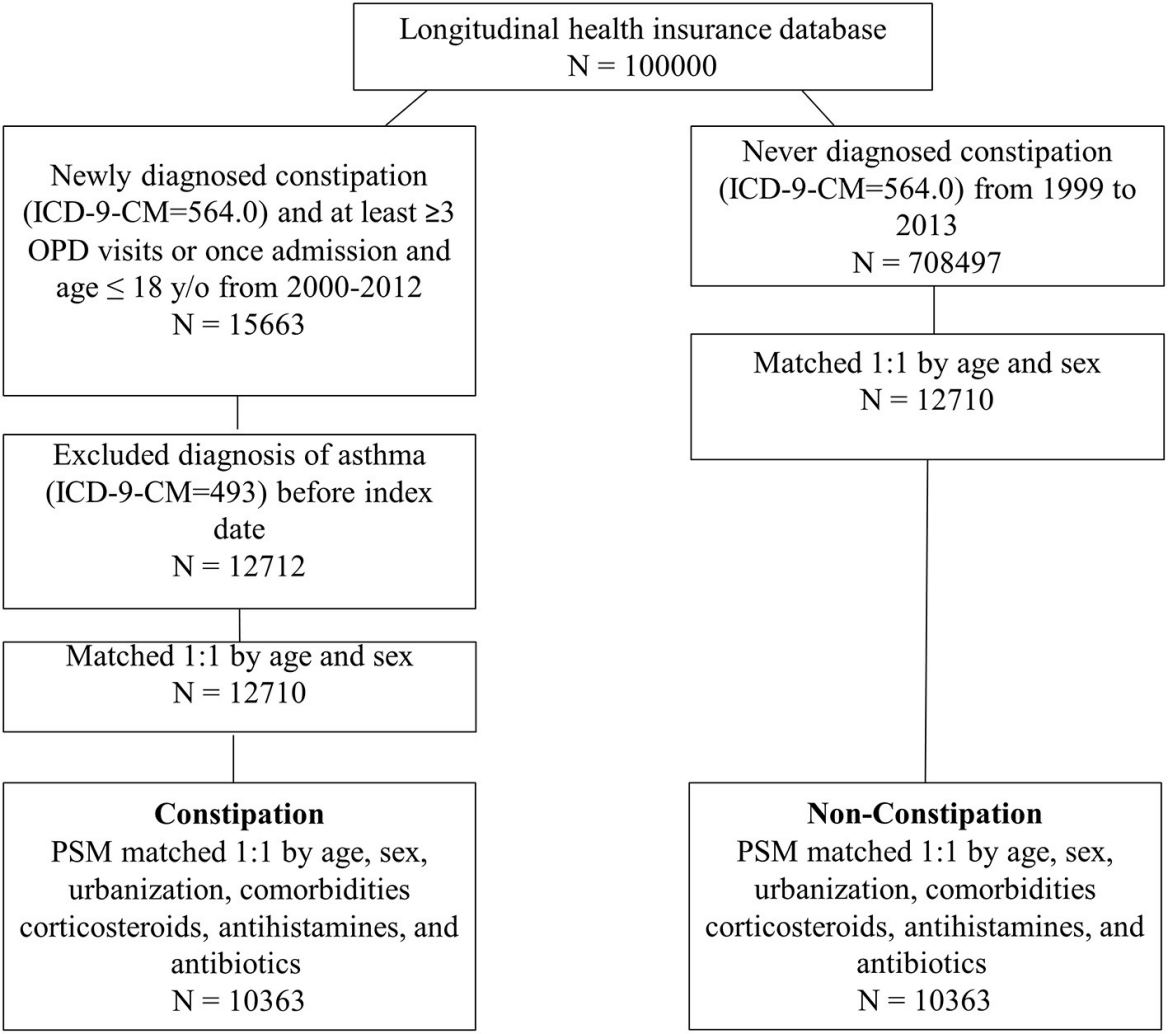
Flowchart of enrolment of constipation and non-constipation groups.

The flowchart is shown in Figure 1. We identified 10,363 patients with constipation and 10,363 matched controls between 1999 and 2013 from the LHID. The demographic characteristics of the study participants are shown in Table 1. The individuals in the constipation and non-constipation cohorts were similar in age and sex distribution. After propensity score matching, there were no statistically significant differences between the constipation and non-constipation groups.

**Table 1:** Demographic characteristics of the constipation group and non-constipation group.

	**Before propensity score matching**			**After propensity score matching**		
	**Constipation (*N* = 12,710)**	**Non-constipation (*N* = 12,710)**	**ASD**	**Constipation (*N* = 10,363)**	**Non-constipation (*N* = 10,363)**	**ASD**
Age (years)			<0.001			0.027
0–6	4,731 (37.2)	4,731 (37.2)		4,157 (40.1)	4,064 (39.2)	
7–12	2,823 (22.2)	2,823 (22.2)		2,206 (21.3)	2,231 (21.5)	
13–18	5,156 (40.6)	5,156 (40.6)		4,000 (38.6)	4,068 (39.3)	
Mean ± SD	9.4 ± 5.8	9.4 ± 5.8	<0.001	9.2 ± 5.9	9.2 ± 5.9	0.004
Sex			<0.001			0.011
Female	8,753 (68.9)	8,753 (68.9)		7,021 (67.8)	7,072 (68.2)	
Male	3,957 (31.1)	3,957 (31.1)		3,342 (32.2)	3,291 (31.8)	
Urbanization			0.081			0.022
Urban	6,868 (54.0)	7,416 (58.3)		5,865 (56.6)	5,801 (56.0)	
Suburban	4,537 (35.7)	4,164 (32.8)		3,538 (34.1)	3,593 (34.7)	
Rural	1,305 (10.3)	1,130 (8.9)		960 (9.3)	969 (9.4)	
Allergic rhinitis	427 (3.4)	177 (1.4)	0.129	205 (2.0)	177 (1.7)	0.020
Allergic conjunctivitis	121 (1.0)	70 (0.6)	0.046	55 (0.5)	62 (0.6)	0.009
Atopic dermatitis	114 (0.9)	58 (0.5)	0.054	44 (0.4)	55 (0.5)	0.015
Bronchiolitis	597 (4.7)	426 (3.4)	0.068	414 (4.0)	418 (4.0)	0.002
Corticosteroids	2,032 (16.0)	1,271 (10.0)	0.179	1,241 (12.0)	1,257 (12.1)	0.005
Antihistamines	10,716 (84.3)	8,963 (70.5)	0.334	8,395 (81.0)	8,379 (100.0)	0.004
Antibiotics	9,790 (77.0)	7,661 (60.3)	0.367	7,503 (72.4)	7,442 (71.8)	0.013

*ASD, Absolute standardized differences*.

Table 2 shows that patients with constipation had a significantly higher risk of asthma than those without constipation after adjustment in the multivariate analysis (aHR: 2.36, 95% C.I. 2.04–2.73, *p* < 0.001). Compared with women, men had a significantly higher risk of asthma (aHR: 1.55; 95% C.I. 1.35–1.77; *p* < 0.001). In term of comorbidities, we observed that people with atopic dermatitis or bronchiolitis had a relatively higher risk of asthma (atopic dermatitis:2.18, 95% C.I. 1.20–3.98, *p* = 0.011; bronchiolitis:1.42, 95% C.I. 1.13–1.79, *p* = 0.003). By contrast, patients using corticosteroids, antihistamines, or antibiotics during the study for a period of at least 30 days had a lower risk of asthma (corticosteroids: 0.79, 95% C.I. 0.64–0.99, *p* = 0.038; antihistamines: 0.33, 95% C.I. 0.27–0.40, *p* < 0.001; antibiotics: 0.38, 95% C.I. 0.32–0.44, *p* < 0.001).

**Table 2:** Cox proportional hazard model analysis for risk of asthma.

	**Univariate**		**Multivariate^**†**^**	
	**HR (95% C.I.)**	***p*-value**	**HR (95% C.I.)**	***p*-value**
Group
Non-constipation	1		1	
Constipation	2.21 (1.91–2.56)	<0.001	2.36 (2.04–2.73)	<0.001
Age (years)
0–6	1		1	
7–12	0.18 (0.14–0.23)	<0.001	0.11 (0.08–0.14)	<0.001
13–18	0.08 (0.06–0.11)	<0.001	0.04 (0.03–0.06)	<0.001
Sex
Female	1		1	
Male	2.33 (2.03–2.66)	<0.001	1.55 (1.35–1.77)	<0.001
Urbanization
Urban	1		1	
Suburban	0.81 (0.70–0.94)	0.007	0.83 (0.71–0.96)	0.014
Rural	0.77 (0.59–0.99)	0.043	0.82 (0.63–1.06)	0.127
Allergic rhinitis	1.29 (0.82–2.03)	0.277	1.50 (0.95–2.37)	0.083
Allergic conjunctivitis	0.22 (0.03–1.54)	0.126	0.42 (0.06–3.01)	0.391
Atopic dermatitis	2.89 (1.59–5.24)	<0.001	2.18 (1.20–3.98)	0.011
Bronchiolitis	2.64 (2.10–3.31)	<0.001	1.42 (1.13–1.79)	0.003
Corticosteroids	0.90 (0.73–1.11)	0.332	0.79 (0.64–0.99)	0.038
Antihistamines	0.91 (0.77–1.08)	0.294	0.33 (0.27–0.40)	<0.001
Antibiotics	0.53 (0.46–0.61)	<0.001	0.38 (0.32–0.44)	<0.001

† *Adjusted for age, sex, urbanization, allergic rhinitis, allergic conjunctivitis, atopic dermatitis, bronchiolitis, corticosteroids, antihistamines, and antibiotics*.

As shown in Table 3, subgroup analyses were performed to assess the association between constipation and asthma based on demographic characteristics. People in the constipation group aged 7–12 years, had a 2.40-fold greater risk of asthma compared with the same age group in the non-constipation group (aHR; 95% C.I. 1.44–4.01, *P* < 0.001). Patients aged 0–6 and 13–18 years in the constipation group had a 2.22-fold and 1.76-fold greater risk of asthma (aHR; 95% C.I. 1.89–2.59, *P* < 0.001; 95% C.I. 1.02–3.03; *P* = 0.042). Among females, compared with those without constipation, there was a 2.62-fold higher risk of asthma in patients with constipation (aHR; 95% C.I. 2.11–3.26; *P* < 0.001). Among males, there was 1.89-fold higher risk of asthma in patients with constipation (aHR; 95% C.I. 1.56–2.30; *P* < 0.001). Furthermore, constipated patients had a higher likelihood of asthma, regardless of urbanization, comorbidities and medications.

**Table 3:** Subgroup analysis of the association between constipation and asthma development.

	**Constipation**		**Non-constipation**			
	***N***	**No. of asthma**	***N***	**No. of asthma**	**HR (95% C.I.)**	***p*-value**
Age (years)						
0–6	4,157	492	4,064	227	2.22 (1.89–2.59)	<0.001
7–12	2,206	49	2,231	21	2.40 (1.44–4.01)	<0.001
13–18	4,000	34	4,068	21	1.76 (1.02–3.03)	0.042
*p* for interaction = 0.611						
Sex						
Female	7,021	289	7,072	115	2.62 (2.11–3.26)	<0.001
Male	3,342	286	3,291	154	1.89 (1.56–2.30)	<0.001
*p* for interaction = 0.031						
Urbanization						
Urban	5,865	339	5,801	180	1.92 (1.60–2.30)	<0.001
Suburban	3,538	183	3,593	76	2.53 (1.94–3.31)	<0.001
Rural	960	53	969	13	4.30 (2.34–7.88)	<0.001
*p* for interaction = 0.019						
Allergic rhinitis						
No	10,158	560	10,186	265	2.19 (1.89–2.54)	<0.001
Yes	205	15	177	4	3.37 (1.12–10.16)	0.031
*p* for interaction = 0.423						
Allergic conjunctivitis						
No	10,308	574	10,301	269	2.21 (1.91–2.55)	<0.001
Yes	55	1	62	0	NA	NA
Atopic dermatitis						
No	10,319	565	10,308	268	2.18 (1.88–2.52)	<0.001
Yes	44	10	55	1	14.08 (1.80–110.07)	0.012
*p* for interaction = 0.069						
Bronchiolitis						
No	9,949	519	9,945	242	2.22 (1.90–2.58)	<0.001
Yes	414	56	418	27	2.22 (1.40–3.52)	<0.001
*p* for interaction = 0.962						
Corticosteroids						
No	9,122	523	9,106	225	2.41 (2.06–2.81)	<0.001
Yes	1,241	52	1,257	44	1.21 (0.81–1.81)	0.347
*p* for interaction = 0.002						
Antihistamines						
No	1,968	123	1,984	34	3.83 (2.62–5.60)	<0.001
Yes	8,395	452	8,379	235	1.97 (1.68–2.31)	<0.001
*p* for interaction = 0.001						
Antibiotics						
No	2,860	240	2,921	93	2.79 (2.20–3.55)	<0.001
Yes	7,503	335	7,442	176	1.93 (1.61–2.32)	<0.001
*p* for interaction = 0.010						

**Figure 2:**
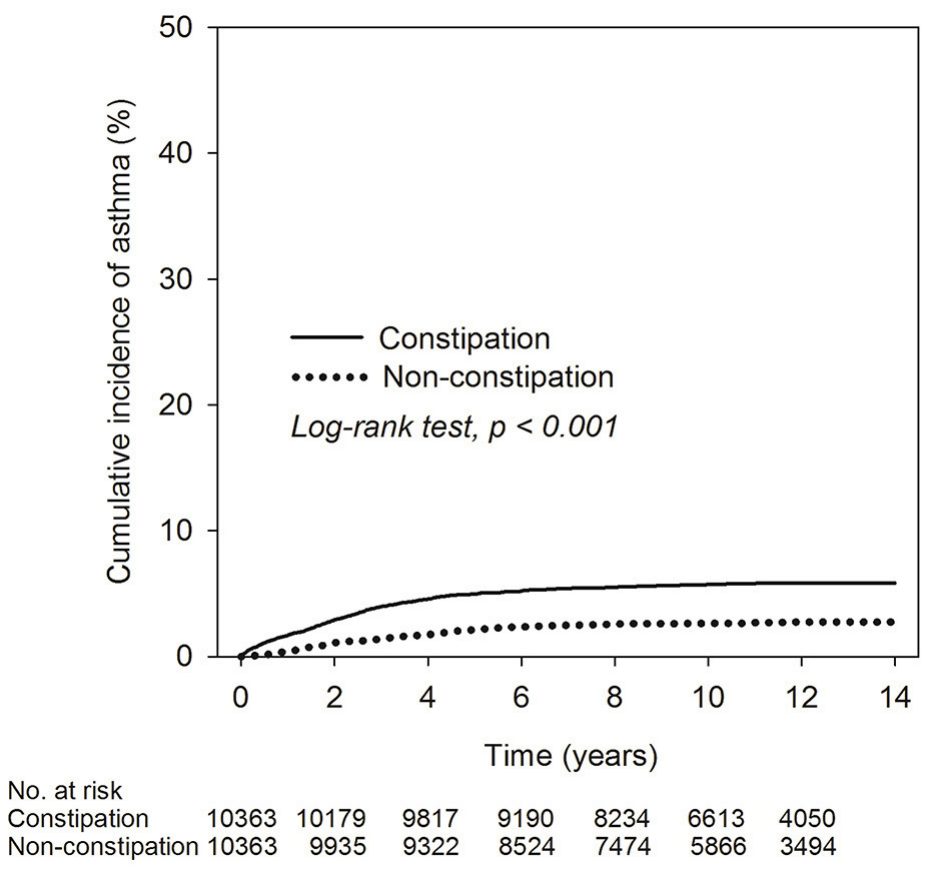
Kaplan–Meier curve of cumulative incidence proportion of asthma in constipation and non-constipation groups.

Table 4 presents the analysis of asthma risk in constipated patients with a prescription of laxatives. Compared to participants without constipation, the adjusted hazard ratio was 2.25-fold (95% C.I. 1.93–2.61; *P* <0.001), 2.85-fold (95% C.I. 2.30–3.53; *P* < 0.001), and 3.44-fold (95% C.I. 1.70–6.96; *P* < 0.001) higher risk of developing asthma in constipated patients with < 3, 3–12, and ≥12 times of laxatives prescription within 1 year, respectively. There appeared to be a dose-effect relationship between constipation severity and risk of asthma. The Kaplan–Meier curves are shown in Figure 2. The cumulative incidence of asthma was significantly higher in constipated patients than in non-constipated patients, and the log-rank test for the comparison of cumulative incidence curves resulted in a *P* < 0.001.

**Table 4:** Cox proportional hazard model analysis for risk of asthma in constipated patients with prescription for laxatives.

	***N***	**No. of asthma**	**Univariate**		**Multivariate^**†**^**	
			**HR (95% C.I.)**	***p*-value**	**HR (95% C.I.)**	***p*-value**
**Group**
Non-constipation	10,363	269	1		1	
Constipation with laxatives prescription <3 times	8,557	442	2.05 (1.76–2.39)	<0.001	2.25 (1.93–2.61)	<0.001
Constipation with laxatives prescription = 3–12 times	1,709	125	2.96 (2.39–3.66)	<0.001	2.85 (2.30–3.53)	<0.001
Constipation with laxatives prescription ≥12 times	97	8	3.45 (1.71–6.96)	<0.001	3.44 (1.70–6.96)	<0.001

† *Adjusted for age, sex, urbanization, allergic rhinitis, allergic conjunctivitis, atopic dermatitis, bronchiolitis, corticosteroids, antihistamines, and antibiotics*.

## Discussion

In this research, children with constipation had a 2.36-fold higher risk for developing asthma than non-constipated individuals, regardless of age, sex, comorbidities, or medications. As far as we know, this is the largest epidemiological study utilizing a nationwide longitudinal population-based dataset to explore the association between constipation and asthma. The findings could be of pathophysiological and clinical importance. Our results highlight the considerably higher risk of asthma in children with constipation. Constipation might be influential in the development of asthma. Pediatricians should be mindful of the possibility of asthma in constipated children. Moreover, constipated patients should be informed of the potential risk of developing asthma and be provided with applicable management for asthma as required. Our findings further highlight the importance of maintaining appropriate bowel habits in order to prevent constipation, which could in turn mitigate risk of asthma.

We also observed that the risk of developing asthma was appreciably increased in children with atopic dermatitis and bronchiolitis. Atopic dermatitis is a chronic inflammatory skin disorder and is regarded as the beginning of the “atopy march” (20), which refers to the natural history of allergic disorders including atopic dermatitis, food allergy (21), asthma, and allergic rhinitis, developing over the course of infancy to childhood. Moreover, some researchers have demonstrated that atopic dermatitis is associated with increased severity of asthma (22, 23). In addition, recent studies revealed a connection between developing asthma and bronchiolitis (24). Our study extends these findings as our results indicate that constipation severity is related to risk of asthma. There seemed to be a “dose-dependent” relationship between constipation and subsequent risk of asthma, which further supports our hypothesis.

The pathophysiological mechanisms underlying the relationship between constipation and asthma remain ambiguous. Recently, there has been considerable research conducted on the link between asthma and gut microbiota (25). The extended “hygiene hypothesis” posits that the initial composition of the infant intestinal microbiota plays a key part in the development of atopic diseases (26, 27). Research conducted in the United Kingdom analyzed the gut microbiome of people with asthma and found that there were abundant *Clostridiums spp.*, whereas *Bacteroides stercoris* and *Faecalibacterium prausnitzii* were depleted in individuals with asthma (28). Similarly, some researches have suggested that alterations in intestinal microflora could lead to constipation and constipation-related symptoms (9). Compared to healthy individuals, constipated patients had relatively higher amounts of potentially pathogenic microbes, such as *Clostridiums spp*. and *Pseudomonas aeruginosa*, and relatively lower amounts of *Lactobacillus, Bacteroides spp*., and *Bifidobacterium* (29). These alterations in the intestinal environment could affect bowel motility by the active metabolites. Some research exhibited that microbial-derived metabolites, predominantly short-chain fatty acids (SCFAs), act as pivotal drivers of T-cell subset activity and proliferation (30). Moreover, it has been found that production of gut microbial SCFAs might down-regulate proinflammatory reactions at the site of allergen insult. Furthermore, SCFAs could affect intestinal motility by stimulating the contraction of colonic smooth muscles, thereby assisting in relief of constipation (31). Another important consideration is the devastating impact of proinflammatory dietary type, or a “Westernized diet,” which might be described as being high content of saturated fats, refined carbohydrates, salt, and reduced consumption of vegetables and fruit (32, 33), on the immune homeostasis, due to change in the intestinal microbiota, resulting in reduced production of SCFAs. Therefore, low fiber intake, such as a diet with little fresh fruit and vegetables, in children with constipation could play a pivotal role in developing asthma (34). The currently available data suggest that the intestinal microbiota might play a key mechanistic role linking asthma and constipation. It is not known how constipation affects the configuration of the bowel microflora and how applicable this condition is to asthma. Nevertheless, constipation seems to be a predisposing factor for asthma. Further comprehensive metabolomic and metagenomic analyses of the intestinal microbiome in constipated children are warranted to clarify the potential mechanisms underlying these associations.

The major advantages of this study were the relatively long follow-up period and the large sample size. An integrated past history of used medical services was accessible for all cases. Therefore, there may have been slight information, selection, and recall bias. As such, it was feasible to properly examine our hypothesis. However, there were still some potential limitations in our study. First, the NHIRD does not include data on covariates, such as mode of delivery, genetic data, personal lifestyle, family history, social adversity, laboratory data, and environmental factors. Even though we adjusted for several comorbidities and matched propensity scores, these unmeasured confounding factors could have influenced our results. Second, the diagnoses of asthma and constipation were dependent on the ICD-9 codes in the administrative dataset. We did not undergo a comprehensive review of the patients' medical records totally so it was not possible to check the accuracy of diagnoses, and thus some misclassifications may have existed. Nevertheless, we tried to increase the accuracy of asthma diagnosis by requiring, in addition to ICD-9 codes, a prescription for an anti-asthmatic medication, as done in the previous studies (35). It is worth noting, however, that any misclassifications were likely to be random, and associations were often underestimated rather than overestimated. In addition, clinical judgment might be different among pediatricians, and so diagnoses would not have been consistent, which might have affected the validity. However, Taiwan's National Health Insurance administration monitors the accuracy of the claims data to prevent violations. Finally, it is unclear as to whether the findings of this study can be extrapolated to other ethnic groups, as the majority of our subjects were Taiwanese. Further clinical research should include other ethnicities and nationalities to determine the generalizability of the relationship observed herein.

In conclusion, constipated children had a 2.36-fold greater risk for asthma compared with those without constipation. Individuals with constipation should be alerted to the elevated risk of developing asthma. Furthermore, in children with asthma, pediatricians should evaluate the patient's bowel condition, including the gut microbiota. The precise pathophysiological association between constipation and asthma requires further research.

## Data Availability Statement

The LHID is a subset of the NHIRD, a database of all medical claims in Taiwan's NHI system. The usage of NHIRD is limited to research purposes only. Only Taiwanese citizens who fulfill the requirements for conducting research projects are eligible to apply for access to the National Health Insurance Research Database (NHIRD). Applicants must follow the Personal Data Protection Act (https://law.moj.gov.tw/ENG/LawClass/LawAll.aspx?pcode=I0050021) and related regulations of the National Health Insurance Administration and NHRI (National Health Research Institutes), and an agreement must be signed by the applicant and his/her supervisor upon application submission. The datasets generated and analyzed during the current study are available from the authors on reasonable request.

## Ethics Statement

The studies involving human participants were reviewed and approved by Institutional Review Board of Chung Shan Medical University Hospital (Approval number CS15134). Written informed consent to participate in this study was provided by the participants' legal guardian/next of kin. Written informed consent was obtained from the individual(s), and minor(s)' legal guardian/next of kin, for the publication of any potentially identifiable images or data included in this article.

## Author Contributions

Y-CH and M-CW drafted the manuscript. JW revised the manuscript critically. All authors provided a substantial contribution to the conception, design, interpretation of the work, and approved the final version of the manuscript.

## Conflict of Interest

The authors declare that the research was conducted in the absence of any commercial or financial relationships that could be construed as a potential conflict of interest.

## Publisher's Note

All claims expressed in this article are solely those of the authors and do not necessarily represent those of their affiliated organizations, or those of the publisher, the editors and the reviewers. Any product that may be evaluated in this article, or claim that may be made by its manufacturer, is not guaranteed or endorsed by the publisher.
